# Spatial-Temporal Variation and Primary Ecological Drivers of *Anopheles sinensis* Human Biting Rates in Malaria Epidemic-Prone Regions of China

**DOI:** 10.1371/journal.pone.0116932

**Published:** 2015-01-22

**Authors:** Zhoupeng Ren, Duoquan Wang, Jimee Hwang, Adam Bennett, Hugh J. W. Sturrock, Aimin Ma, Jixia Huang, Zhigui Xia, Xinyu Feng, Jinfeng Wang

**Affiliations:** 1 State Key Laboratory of Resources and Environmental Information System, Institute of Geographic Science and Natural Resource Research, Chinese Academy of Sciences, Beijing, China; 2 University of Chinese Academy of Sciences, Beijing, China; 3 Key Laboratory of Surveillance and Early Warning on Infectious Disease, Chinese Center for Disease Control and Prevention, Beijing, China; 4 National Institute of Parasitic Diseases, Chinese Center for Disease Control and Prevention, WHO Collaborating Center for Malaria, Schistosomiasis and Filariasis, Key Laboratory of Parasite and Vector Biology, Ministry of Health, Shanghai, People’s Republic of China; 5 Malaria Elimination Initiative, Global Health Group, University of California San Francisco, San Francisco, California, United States of America; 6 Malaria Branch, Division of Parasitic Diseases and Malaria, Centers for Disease Control and Prevention, Atlanta, Georgia, United States of America; 7 College of Geoscience and Surveying Engineering, China University of Mining and Technology, Beijing, China; 8 Center of 3S Technology and Mapping, Beijing Forestry University, Beijing, China; Centers for Disease Control and Prevention, UNITED STATES

## Abstract

**Background:**

Robust malaria vector surveillance is essential for optimally selecting and targeting vector control measures. Sixty-two vector surveillance sites were established between 2005 and 2008 by the national malaria surveillance program in China to measure *Anopheles sinensis* human biting rates. Using these data to determine the primary ecological drivers of malaria vector human biting rates in malaria epidemic-prone regions of China will allow better targeting of vector control resources in space and time as the country aims to eliminate malaria.

**Methods:**

We analyzed data from 62 malaria surveillance sentinel sites from 2005 to 2008. Linear mixed effects models were used to identify the primary ecological drivers for *Anopheles sinensis* human biting rates as well as to explore the spatial-temporal variation of relevant factors at surveillance sites throughout China.

**Results:**

Minimum semimonthly temperature (*β* = 2.99; 95% confidence interval (CI) 2.07- 3.92), enhanced vegetation index (*β* =1.07; 95% CI 0.11–2.03), and paddy index (the percentage of rice paddy field in the total cultivated land area of each site) (*β* = 0.86; 95% CI 0.17–1.56) were associated with greater *An. Sinensis* human biting rates, while increasing distance to the nearest river was associated with lower *An. Sinensis* human biting rates (*β* = −1.47; 95% CI −2.88, −0.06). The temporal variation ($\sigma_{t0}^{2}\;=\;1.35$) in biting rates was much larger than the spatial variation ($\sigma_{s0}^{2}\;=\;0.83$), with 19.3% of temporal variation attributable to differences in minimum temperature and enhanced vegetation index and 16.9% of spatial variance due to distance to the nearest river and the paddy index.

**Discussion:**

Substantial spatial-temporal variation in *An. Sinensis* human biting rates exists in malaria epidemic-prone regions of China, with minimum temperature and enhanced vegetation index accounting for the greatest proportion of temporal variation and distance to nearest river and paddy index accounting for the greatest proportion of spatial variation amongst observed ecological drivers.

**Conclusions:**

Targeted vector control measures based on these findings can support the ongoing malaria elimination efforts in China more effectively.

## Introduction

In China, *Anopheles sinensis* is an important malaria vector with the largest geographic distribution, being present between 25°N and 33°N latitude. *An. sinensis* is an outdoor biting and resting mosquito, which breeds in a wide variety of water collections and has a number of potential resting sites, including rice fields, straw heaps, and low vegetation [1]. Although a relatively inefficient vector because of its zoophilic habits, *An. sinensis* is still considered an important vector of *Plasmodium vivax* malaria in China due to its wide distribution and high density [2–4].

Many current malaria control and elimination interventions aim to reduce human-vector contact [5, 6]. In China, malaria vector surveillance has been conducted recently in malaria epidemic-prone regions to gain a basic understanding of transmission parameters, assess impact of insecticide-based control measures, and identify receptive areas for malaria transmission [7, 8]. Moreover, recent surveillance results have indicated a high level of heterogeneity in *An. sinensis* distribution throughout epidemic-prone regions where *An. sinensis* biting rates averaged 6.2 bites per man per night, but ranged from 0.4 to 107 bites per man per night [9].

It is well known that malaria infections are not distributed homogenously, with some areas within the same region showing higher incidence than others [10]. Many factors may contribute to the spatial heterogeneity of transmission intensity in a community, including the distance to larval habitats, land cover, topography, and presence of livestock [11–13]. Malaria outbreaks and re-emergences in recent years in China have only occurred in regions where *An. sinensis* was the primary vector [14], but the ecological factors influencing *An. sinensis* abundance in China are poorly understand. Previous studies looking at *An. sinensis* density have only examined a limited set of climatic factors such as temperature and precipitation [15]. Few studies have considered additional meteorological and socioeconomic factors when exploring the spatial-temporal variation of *An. sinensis* density. Understanding the associated drivers for spatial-temporal dynamics of *An. sinensis* biting rates is crucial to the development of effective malaria elimination measures in China.

An important task of disease vector ecology research is to determine the relative contribution of related ecological factors on spatial-temporal heterogeneity of the vector distribution. The use of remote sensing (RS), geographic information systems (GIS), and spatial statistics in the study of vector-borne diseases has increased remarkably during recent years [16, 17]. This has been especially true for studies of anopheline mosquitoes whose dependence on water in early stages of life cycle makes them particularly amenable to study by RS. Several studies have used low-resolution satellite imagery to monitor the climatic factors associated with malaria transmission [17–19]. These models tend to result in good predictions over large areas, where the mosquito dynamics are mainly driven by rainfall and temperature patterns.

In this evaluation, we analyzed data from China’s large national vector surveillance program. We used linear mixed effects models to explore the relative contribution of primary ecological drivers for spatial-temporal variation of *An. sinensis* in malaria epidemic-prone regions of China. Both time-invariant and time-variant factors were included in the model simultaneously to explore their respective contribution to spatial-temporal variation in *An. sinensis* human biting rate.

This is the first study in China presenting a systematic analysis of ecological drivers (including socioeconomic, climatic, environmental factors) of the spatial-temporal distribution of *An. sinensis* throughout the country, which provides an invaluable guide for targeting vector control measures to support the ongoing malaria elimination program.

## Methods

### Study areas

**Figure 1:**
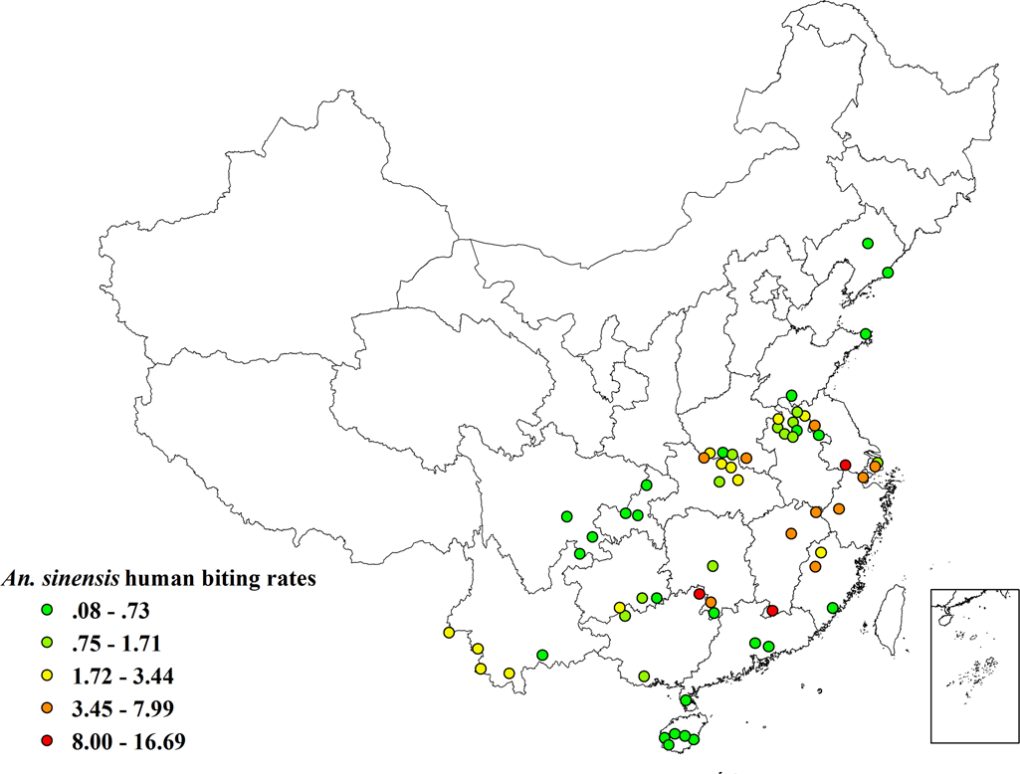
The distribution of *Anopheles sinensis* human biting rates averaged by each monitoring site during 2005 to 2008 in China.

This evaluation included entomological data from 62 malaria surveillance sites established between 2005 and 2008 by the national malaria sentinel surveillance program in China (Fig. 1). Each site is comprised of a township, which typically has a population of 10,000–30,000 and is comprised of 40–100 natural villages. Sentinel townships were divided into the following three categories based on the transmission levels in China [1]:

1. Unstable endemic areas: 30 townships in 6 provinces (Hainan, Yunnan, Anhui, Hubei, Henan, Jiangsu) [5 counties/ province, 1 township/county].
2. Low endemic areas: 24 townships in 8 provinces (Sichuan, Chongqing, Guizhou, Guangdong, Guangxi, Hunan, Jiangxi, Fujian) [3 counties/province, 1 township/county].
3. Pre-elimination areas: 8 townships in 4 provinces (Shanghai, Zhejiang, Shandong, Liaoning) [2 counties/province, 1 township/county].

### Ethical considerations

Ethics approval was obtained from the National Institute of Parasitic Disease, Chinese Center for Disease Control and Prevention (WHO Collaborating Center for Malaria, Schistosomiasis and Filariasis) ethical committee. No specific permissions were required for these activities, the location is not privately owned and the field studies did not involve endangered or protected species.

### Mosquito collection

Based on the malaria prevalence during the past five years as well as ecological variation, one representative natural village from each sentinel surveillance township was selected for the routine vector surveillance for malaria. Based on WHO recommendations [21], outdoor human landing catches were made by two adult volunteers from the local population working beside a bed net with one sleeping person. Mosquitoes coming to bite the collectors or sleeping person were detected using a flashlight, collected using glass tubes with backpack aspirator (CDC backpack aspirator: John W. Hock Co., Florida, USA) and placed in the screened pint-sized containers. Collections were conducted for 30 min each hour from 18:00 to 06:00 every 15 days from June to October, 2005–2008. Collectors worked in pairs in 6 hour shifts. One pair began at 18:00 and another at midnight. Mosquitoes were taken to provincial laboratory and killed by suffocation with chloroform vapor. They were counted as well as identified morphologically using taxonomic keys [7]. The mosquito human biting rate was calculated as the number of female adults landing on humans per house man-hour.

### Meteorological and environmental variables

**Figure 2:**
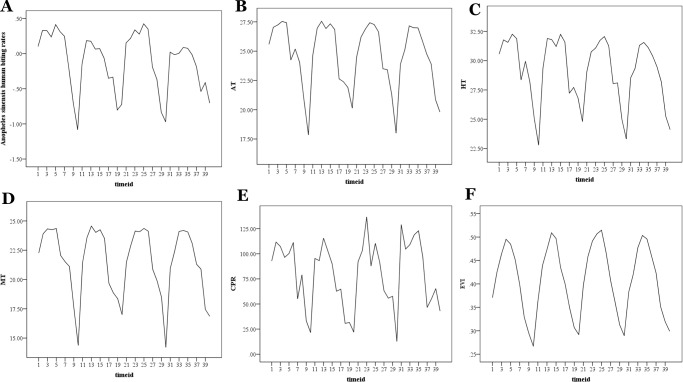
Time series plots of *Anopheles sinensis* human biting rates and time-variant predictors. (A) *Anopheles sinensis* human biting rates (Box-cox transformed); (B) average temperature [AT]; (C) highest temperature [HT]; (D) minimum temperature [MT]; (E) cumulative precipitation [CPR]; (F) enhanced vegetation index [EVI]. The timeid in this figure indicates the order number of semimonth from June 2005 to October 2008. For example, 1 indicates the first half of June 2005; 11 indicates the first half of June 2006.

Based on previous studies demonstrating that fluctuations in anopheline abundance are driven primarily by temperature and precipitation [13, 22, 23], four meteorological variables (average temperature [AT], highest temperature [HT], minimum temperature [MT] and cumulative precipitation [CPR]), measured every day at 680 stations and aggregated to semimonthly intervals for each station were used to examine the relationship between climate and *An. sinensis* human biting rates in our study. Meteorological data were collected from the publicly available Chinese Meteorological Data Sharing Service System (http://cdc.cma.gov.cn/home.do). The meteorological records in 40 time points (semimonthly, June to October from 2005 to 2008) at 680 stations were interpolated separately for each time period across all sites by using Inverse Distance Weighting interpolation technique in ArcGIS 10.1 (Environmental Systems Research Institute, Redlands, California, USA). All these meteorological variables were normalized using the min-max normalization method, in order to adjust values measured on different scales to a common scale [24]. Fig. 2 gives the time-series plots of these meteorological variables.

Several recent studies [25, 26] have shown significant correlations between the vegetation indices derived from Moderate-resolution Imaging Spectroradiometer (MODIS) Terra satellite and mosquito density. Vegetation indices could relate to surface moisture and presence of vegetation types which are naturally around where vectors are found [25]. Here, the 16-day composite MODIS Normalized Difference Vegetation Index (NDVI) as well as Enhanced Vegetation Index (EVI) at a resolution of 250 meters (https://lpdaac.usgs.gov/products/modis_products_table/mod13q1) was used to explain the variation of *An. sinensis* human biting rates. Normalized Difference Vegetation Index values vary between +1 and -1; the higher the NDVI value, the denser the green vegetation [27]. EVI performs better than NDVI in dense vegetation coverage areas because of the atmospheric and background corrections incorporated into EVI’s calculation [28]. Since some studies [14, 29, 30] have found that distance to water bodies is negatively associated with mosquito density, we generated raster maps depicting the distance of every pixel to the nearest river by applying straight line distance interpolation function in ArcGIS 10.1 [31]. Landform (plain, mountain, hill and basin), slope and elevation were also explored as potential predictor variables.

### Socioeconomic variables

Socioeconomic factors including the number of livestock and paddy index were explored for inclusion in models based on previous evidence of their importance in China [13, 32]. Paddy index reflects the relative amount of land devoted to rice cultivation, and is defined as the rice paddy field area divided by the total cultivated land area at township level according to the National Statistical Bureau; this proportion was used to describe the potential breeding environment for mosquitos.

### Categories of predictor variables

The analyzed variables were divided into two categories: the time-variant and time-invariant factors. The time-invariant covariates included river distance, paddy index, landform, livestock, slope and elevation which are time-invariable or change extremely slowly over time. The time-variant covariates AT, HT, MT, NDVI and EVI were measured semimonthly during the surveillance period.

### Mixed effects model

Mixed effects models, also called hierarchical models, random-effects, or random-coefficient models, have been widely used in various fields to explore determinants of spatial-temporal variation of a number of outcomes [33]. There are three advantages to mixed effects models compared to simple regression models: (1) they are robust to missing data and irregularly spaced measurement occasions [34]; (2) they are able to incorporate correlation structures that often exist within grouped data [35]; and (3) the groups can be treated as random effects to model the covariance structure introduced by the grouping of the data. By using a mixed effects model, we can deal with the potential unobserved heterogeneity among surveillance sites.

**Figure 3:**
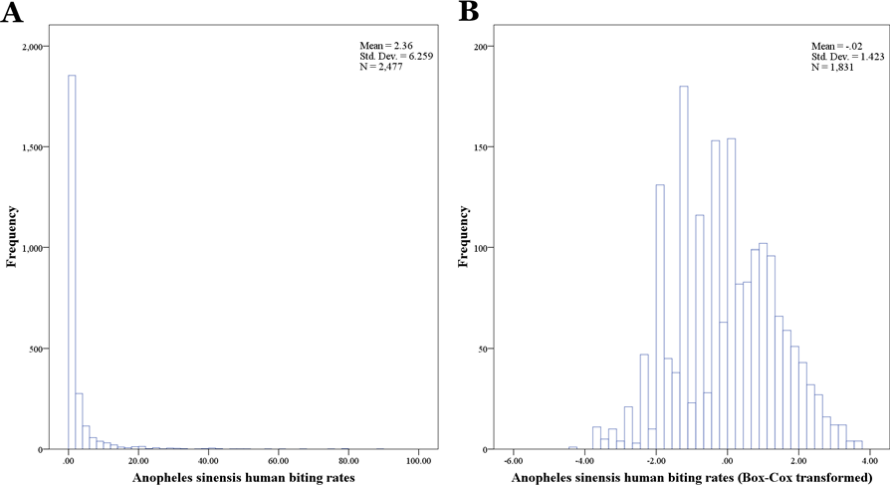
The histogram of the original (A) and Box-cox (B) transformed *Anopheles sinensis* human biting rates.

In this study, mixed effects models were implemented using the *xtmixed* command in the statistical software Stata 12 (College Station, Texas) [36]. In order to meet the Gaussian assumption of normally-distributed residuals, a Box-Cox transformation was used to transform the raw *An. sinensis* human biting rates data [37]. Fig. 3 shows the histogram of original and Box-Cox transformed *An. sinensis* human biting rates.

### Mixed effects model to analyze mosquito human biting rate

Mixed effects models were used to examine the relationship between *An. sinensis* human biting rates and predictor variables. We used two types of mixed effects models: the variance component model and random intercept model [24, 38].

In order to examine the spatial and temporal variation of *An. sinensis* human biting rates, a variance component model (equation 1) was used to fit the data. A variance component model is an “empty” model that does not include any explanatory variables but only estimates the spatial and temporal differences in *An. sinensis* human biting rates [24].

$$y_{it}=\alpha_{i}+\nu_{i}+u_{it}\;i=1,\cdots ,N;t=1,\cdots ,T_{i}$$ (1)

Where *y_it_* is the *An. sinensis* human biting rate in *i*-th monitoring site (township) and *t-th* semimonth; where *ν_i_* is the difference between average *An. sinensis* human biting rate in each site and global average *An. sinensis* human biting rate among all sites; *u_it_* is the difference between *y_it_* and average *An. sinensis* human biting rate in each site; $\sigma_{\nu }^{2}$ and $\sigma_{u}^{2}$ capture spatial and temporal variation of *An. sinensis* human biting rates, respectively.

Equation 1 can be specified using a variety of assumptions about spatial heterogeneity of the relation between independent variables and dependent variable. If only intercepts vary (*α_i_*) among surveillance sites, fixed/ random effects estimates can be used to estimate equation 2.

$$y_{it}=\alpha_{i}+\beta X_{it}+\nu_{i}+u_{it}\text{i}=1,\cdots ,N;\text{t}=1,\cdots ,T_{i}$$ (2)

Equation 2 assumes that the error term is serially uncorrelated conditional on the individual effect *α_i_*. However, unobserved variables varying systematically over time may violate this assumption [39]. To provide more general autocorrelation scheme, one can relax the restriction that *u_it_* follow a first-order autoregressive process [39],

$$u_{it}=\rho_{i,t-1}+\eta_{it}$$ (3)

where *ρ* is the serial correlation coefficient; ∣*ρ*∣ < 1 and *η_it_* is independent and identically distributed (i.i.d.) with mean 0 and variance $\sigma_{\eta }^{2}$;

The Lagrange-Multiplier test was used to test whether there was significant serial correlation. A likelihood ratio test was used to test spatial heterogeneity by comparing the random intercept model with single level regression model.

Previous studies have showed that meteorological variables with 1–2 month lag were significantly associated with malaria incidence in China [40, 41]; we used mixed effects model to explore the lag effects of time-variant factors on *An. sinensis* human biting rates.

### Proportional change in variance (PCV) for spatial and temporal variation

The spatial and temporal variation of *An. sinensis* human biting rate can be attributed to different factors including time-variant factors (e.g., MT, CPR and EVI) and time-invariant factors (e.g., elevation, slope, paddy index). By adjusting for time-variant factors in a random intercept model, we can calculate the proportional change in temporal variance (PCV_t_) by each time-variant factor [24, 42]. The PCV_t_ can measure how much temporal variance can be explained by each time-variant factor. The equation for the proportional change in temporal variance (PCV_t_) of *An. sinensis* human biting rate can be written as:

$${\text{PCV}}_{\text{t}}=\frac{{\text{V}}_{\text{t}0}-{\text{V}}_{\text{t}1}}{{\text{V}}_{\text{t}0}}$$ (4)

Where *V_t_*_0_ is the temporal variation in variance component; and *V_t_*_1_ is the temporal variation in the model including time-variant factors. The equation can be adapted to calculate the PCV at spatial dimension (PCV_s_), as variance in *An. sinensis* human biting rate among surveillance sites which will also be explained by differences in the time-invariant factors used in the study.

$${\text{PCV}}_{\text{s}}=\frac{{\text{V}}_{\text{s}0}-{\text{V}}_{\text{s}1}}{{\text{V}}_{\text{s}0}}$$ (5)

## Results

### Mosquito collections

A total of 35,859 female *An. sinensis* were captured from the surveillance sites during 2,480 nights of collecting from 2005 to 2008. There was a significant difference in distribution and human biting rate of *An. sinensis* across the surveillance sites (Fig. 1) and a seasonal peak of abundance each year in the rainy season (July-August) (Fig. 2A). The biting rate of *An. sinensis* per site averaged 2.48 bites per man per night and ranged from 0.08 to 16.70 bites per man per night. The three highest biting rates were observed at Quanzhou site (16.69 bites per man per night) in Guangxi Province, followed by Yixin (16.03 bites per man per night) in Jiangsu Province and Longnan (10.79 bites per man per night) in Jiangxi Province, while the three lowest rates were found at Weihai (0.08 bites per man per night) in Shandong Province, Jiangyang (0.12 bites per man per night) in Sichuan Province and Wanning (0.15 bites per man per night) in Hainan Province.

### Model evaluation

**Figure 4:**
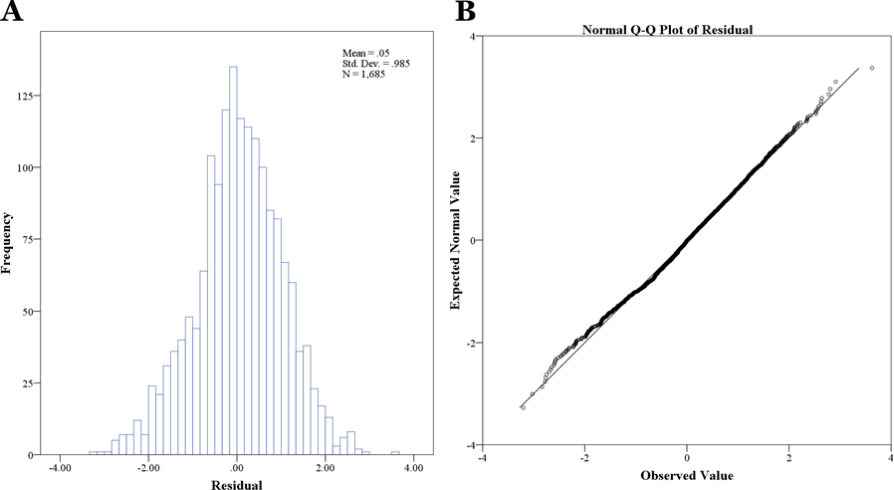
Checking the residuals for multivariate analysis. A. histogram of residuals. B. Q-Q plot of residuals.

The Lagrange-Multiplier test (F = 65.3, *df* = 60, P<0.001) showed that significant serial correlation and first-order autoregressive structure (AR1) should be used in the error term. A likelihood ratio test indicated that all random intercept models were appropriate over single level regression models (Table 1, 2, 3). Fig. 4 shows that the residuals derived from multivariate analysis indicate good performance of our model.

**Table 1:** Variance component model.

	**Estimate (95% Confidence Interval)**
***Fixed effects***	
**Constant**	-0.18 (-0.42, 0.06)
***Random effects***	
${\text{σ}}_{s0}^{2}$	0.83 (0.55,1.24)
${\text{σ}}_{t0}^{2}$	1.35 (1.23,1.49)
*ρ*	0.61 (0.57,0.65)
***LR test***	1371.5 (P<0.001)
***AIC***	5123.4

$\sigma_{s0}^{2}$ indicates spatial variation; $\sigma_{t0}^{2}$ indicates temporal variation; *ρ* is the serial correlation coefficient; LR test indicates likelihood ratio test for monitoring site effects; AIC indicates Akaike Information Criterion.

**Table 2:** The effects of time-variant and time-invariant covariates on *An. sinensis* human biting rates.

***Fixed effects***	***Estimate(95% CI)***	***Random effects***		***AIC***	***LR test***
		${\text{σ}}_{s0}^{2}$	${\text{σ}}_{t0}^{2}$		
**MT**	3.56(2.84, 4.29)*	0.90(0.62, 1.30)	1.10(0.97, 1.26)	4953.5	1493.4**
**CPR**	0.56(0.15, 0.97)*	0.77(0.52, 1.14)	1.25(1.09, 1.44)	5113.7	1383.1**
**EVI**	2.88(1.98, 3.77)*	0.86(0.59, 1.28)	1.15(1.01, 1.32)	5030.4	1453.5**
**Landform**		0.79(0.53, 1.20)	1.35(1.23, 1.46)	5121.0	1363.6**
**Plain** ^#^					
**Mountain**	-0.10(-0.25, 0.46)				
**Hill**	0.05(-0.52, 0.62)				
**Basin**	0.46(0.18, 0.74)*				
**Livestock**	0.23(-0.75, 1.21)	0.74(0.49, 0.63)	1.35(1.23, 1.46)	5125.2	1370.7**
**Slope**	-0.19(-0.88, 0.48)	0.74(0.49, 1.11)	1.35(1.23, 1.49)	5125.3	1364.5**
**River Distance**	-1.17(-2.18, -0.16)*	0.74(0.49, 1.13)	1.35(1.23, 1.48)	5121.2	1309.1**
**Elevation**	0.23(-0.44, 0.90)	0.74(0.50, 1.11)	1.35(1.23, 1.49)	5125.2	1370.6**
**Paddy Index**	1.16(0.49, 1.83)*	0.71(0.50, 1.09)	1.35(1.23, 1.48)	5110.2	1367.5**

* p<0.05,

** p<0.001.

^#^ Reference category.

Abbreviations: MT- minimum temperature; CPR- cumulative precipitation; EVI- enhanced vegetation index.

**Table 3:** Multivariate analysis.

***Fixed effects***	**Estimate (95% Confidence Interval)**
**MT**	2.99 (2.07, 3.92)*
**CPR**	0.15 (-0.35, 0.66)
**CPR_lag1**	0.08 (-0.31, 0.48)
**CPR_lag2**	-0.14 (-0.46, 0.18)
**CPR_lag3**	0.17 (-0.14, 0.48)
**EVI**	1.07 (0.11, 2.03)*
**Landform**	
**Plain^#^**	
**Mountain**	-0.16 (-0.71, 0.39)
**Hill**	0.03 (-0.42, 0.49)
**Basin**	0.49 (-1.58, 2.57)
**River Distance**	-1.47 (-2.88, -0.06)*
**Paddy Index**	0.86 (0.17, 1.56)*
***Random effects***	
${\text{σ}}_{s0}^{2}$	0.69 (0.45, 1.04)
${\text{σ}}_{t0}^{2}$	1.09 (0.99, 1.20)
ρ	0.55 (0.51, 0.60)
**LR test**	1338.1 (P<0.001)
PCV_*t*_(%)	19.3
PCV_*s*_(%)	16.9

Abbreviations: MT- minimum temperature; CPR- cumulative precipitation; EVI- enhanced vegetation index; LR test- likelihood ratio test. PCV_*t*_: Proportional change in temporal variance; PCV_*s*_: Proportional change in spatial variance.

### Spatial and temporal variation

Table 1 shows the spatial-temporal variation of *An. sinensis* human biting rates in China from 2005 to 2008 from a total of 62 sites over 40 time points which were utilized to construct the panel model. According to the variance component model, the temporal variation ($\sigma_{t0}^{2}\;=\;1.35$) of *An. sinensis* human biting rates was more than 1.5 times larger than the spatial variation ($\sigma_{s0}^{2}\;=\;0.83$).

### Univariate analysis

**Figure 5:**
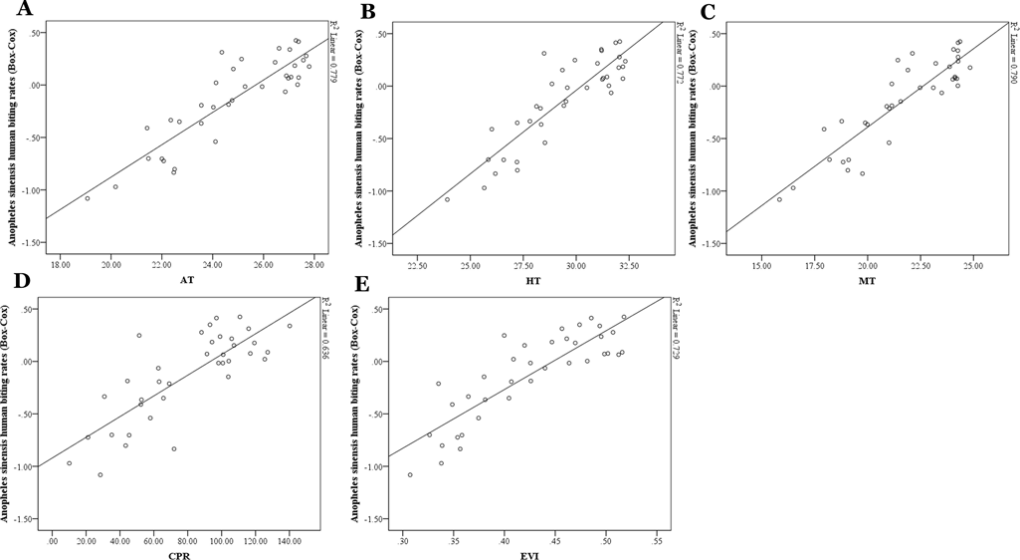
Scatterplots of *Anopheles sinensis* human biting rates and time-variant predictors. *Anopheles sinensis* human biting rates (Box-cox transformed) against (A) average temperature [AT]; (B) highest temperature [HT]; (C) minimum temperature [MT]; (D) cumulative precipitation [CPR]; (E) enhanced vegetation index [EVI].

All the related variables, including the four meteorological variables (AT, HT, MT and CPR) and two vegetation indices (NDVI and EVI), were used in the univariate analysis. Scatterplots suggested that there were plausible linear relationships between *An. sinensis* human biting rate and these time-variant predictors (Fig. 5). In order to avoid collinearity between these variables, MT, CPR and EVI were selected for further analysis by Akaike Information Criterion (AIC) derived from all the univariate analyses (results not shown). All significant variables in Table 2 were used in the multivariate analysis (Table 3). Table 2 shows the relationship between *An. sinensis* human biting rate and predictor variables from the mixed effects model.

For time-variant factors, higher MT was associated with higher *An. sinensis* human biting rate (β = 3.56; 95% CI 2.84 − 4.29). Similar to MT, a significant positive association between precipitation and *An. sinensis* human biting rate (β = 0.56; 95% CI 0.15 − 0.97) was observed. It was also found that *An. sinensis* biting rate increased (β = 2.88; 95% CI 1.98 − 3.77) with increasing EVI. Table 4 shows that only CPR has 1–3 semimonthly lag effects on *An. sinensis* human biting rate, while MT and EVI had no lag effects. This suggests that temperature has short effects, while precipitation may have relatively delayed effects on *An. sinensis* human biting rate.

**Table 4:** The semimonthly lag effects of time-variant covariates on *Anopheles sinensis* human biting rates.

**Lag effects**	**MT**	**CPR**	**EVI**
Lag0	3.59 (2.72, 4.47) *	0.99 (0.44, 1.56) *	2.48 (1.25, 3.71) *
Lag1	-0.09 (-0.71, 0.54)	0.82 (0.38, 1.26) *	0.59 (-0.36, 1.54)
Lag2	-0.22 (-0.65, 0.21)	0.59 (0.28, 0.92) *	-0.14 (-0.85, 0.57)
Lag3	0.14 (-0.34, 0.62)	0.49 (0.16, 0.83) *	-0.19 (-0.96, 0.57)
Lag4	-0.28 (-0.76, 0.21)	-0.06 (-0.48, 0.35)	-0.28 (-1.09, 0.53)

* p<0.05.

Abbreviations: MT- minimum temperature; CPR- cumulative precipitation; EVI- enhanced vegetation index.

For time-invariant factors, *An. sinensis* biting rate in the basin regions was higher than that of plain regions (β = 0.46; 95% CI 0.18 − 0.74). In addition, lower *An. sinensis* human biting rate was found in the regions farther from water bodies than in regions closer to water bodies (β = −1.17; 95% CI − 2.18, −0.16) (Table 2). Sites with a higher paddy index were also more likely to have higher *An. sinensis* human biting rate (*β* = 1.16; 95% CI 0.49 − 1.83). Livestock, elevation and slope were not significantly associated with *An. sinensis* human biting rate.

### Attribution of spatial and temporal variation

Table 5 shows the proportion of spatial and temporal variation explained by the different factors. The inclusion of time-invariant factors did not decrease residual temporal variation, but reduced the spatial variation more or less in terms of whether the coefficients significantly differed from 0 as was expected (Table 2). For example, inclusion of paddy index decreased the residual variance across surveillance sites from 0.83 to 0.71, while the temporal variation did not change. The largest proportion of spatial variation of *An. sinensis* human biting rate was explained by variation in paddy index (14.5%), whereas the landform and river distance explained 4.8% and 10.8% of the variation across surveillance sites, respectively.

**Table 5:** Proportional change in variance at spatial and temporal dimensions.

**Covariates**	**PCV_*t*_ (%)**	**Covariates**	**PCV_*s*_ (%)**
MT	18.5	Landform	4.8
CPR	7.4	River Distance	10.8
EVI	14.8	Paddy index	14.5

PCV_*t*_: Proportional change in temporal variance.

PCV_*s*_: Proportional change in spatial variance.

Abbreviations: MT- minimum temperature; CPR- cumulative precipitation; EVI- enhanced vegetation index.

Similarly, time-variant factors explained the temporal variation other than spatial variation of *An. sinensis* human biting rate. Table 5 indicates MT explained the majority of the temporal variation (PCVt = 18.5%) of *An. sinensis* density in the variance component model, while only 7.4% of the temporal variation was attributable to CPR. However, the EVI explained 14.8% of the temporal variation, indicating that EVI was a better index to model temporal changes in *An. sinensis* human biting rate than CPR.

### Multivariate analysis

Six ecological factors were included in the multivariate mixed effects model based on the univariate analyses (Table 2). For time-variant factors, the multivariate analysis (Table 3) indicated that MT and EVI had a substantial effect (coefficient is *β* = 2.99; 95% CI 2.07 − 3.92 and *β* = 1.07 95% CI 0.11 − 2.03, respectively) on *An. sinensis* human biting rate. Although CPR was an important factor for *An. sinensis* human biting rate in the univariate analysis, the relationship was not significant in the multivariate model (Table 3). In addition, there was no lag effect of CPR according to multivariate analysis, despite relatively long lag effects in the univariate analysis (Table 4). For time-invariant factors, a significant negative association between river distance and human biting rate (*β* = −1.47; 95% CI −2.88, −0.06) was observed, while a significant positive association with paddy index (*β* = 0.86; 95% CI 0.17 − 1.56) was found.

After taking into account time-variant factors (MT and EVI), Table 3 shows 19.3% of the temporal variance of *An. sinensis* human biting rate in the variance component model was attributable to differences in MT and EVI, with 16.9% of the spatial variance due to time-invariant factors including river distance and paddy index.

## Discussion

In this study, we analyzed data from China’s national vector surveillance program to assess the association between *An. sinensis* human biting rates and various ecological predictors. While previous research [13] has explored ecological associations with *An. sinensis* human biting rate over smaller scales, this study explores the influence of socioeconomic, environmental and climatic factors at a country-wide level in China. Moreover, this study proposes a simple approach to estimate the effects of time-variant and time-invariant factors on *An. sinensis* human biting rate using a mixed effects model. This approach can help researchers understand the influence of different types of factors on *An. sinensis* human biting rates.

Importantly, we have identified key ecological factors responsible for *An. sinensis* human biting rate that are of major epidemiological significance. This information can be used to make spatial and temporal predictions, facilitating targeted interventions. Minimum temperature, EVI and paddy index had significant positive effects on the human biting rate of *An. sinensis*, while a significant negative association between river distance and *An. sinensis* human biting rate was observed. The study found that the temporal variation ($\sigma_{t0}^{2}\;=\;1.35$) of *An. sinensis* human biting rate was larger than the spatial variation ($\sigma_{s0}^{2}\;=\;0.82$) based on the variance component model, and 14.1% of the temporal variation of *An. sinensis* human biting rate was attributable to the differences in MT and EVI, while 15.8% of the spatial variance was due to the river distance and the paddy index in the surveillance sites in China.

Over a large geographic scale, this study suggests that human biting rate of *An. sinensis* is mainly driven by climatic factors and environmental factors such as MT and EVI as well as paddy index. Several studies from Africa [43–45] noted that the temporal variation of malaria vectors varied with seasonal variations, while temperature did not have a simple linear relationship with malaria vector human biting rate: within a certain range of temperature, malaria vector human biting rate increases with temperature, while extreme low and high temperature decreases the rate.

The spatial variation in *An. sinensis* human biting rate was attributed to environmental heterogeneity including distance from a river as well as paddy index. In China, numerous studies [13, 32, 46] have shown that the primary habitat of *An. sinensis* is rice fields and the related irrigation system. The survey by Chen and Yang [47] demonstrated that rice fields constituted about 93% of breeding sites for *An. sinensis* in some regions. Similarly, other studies found that the distance of sample sites to rice fields was an important factor in China [1, 48]. The association between distance from the river and mosquito density is consistent with observations from other malaria settings, such as Cameroon, where the main malaria vectors are particularly associated with river beds [49].

This study also found that human biting rate of *An. sinensis* was significantly related to EVI, although these relationships have seldom been elucidated until now. A plausible explanation may be that EVI is a surrogate for more availability of suitable larval habitats of *An. sinensis* in China.

Although the amount of rainfall is a well-known factor related to presence and survival of malaria vectors [50, 51], no clear association was observed between rainfall and *An. sinensis* human biting rate in this study. The difference between our findings, and those of others, may be due to topography or climatologic differences. Equally, the pattern of rainfall might be more important than the amount of rainfall, as light, infrequent rains seem to be most favorable for larval development [52, 53].

As this study suggests that *An. sinensis* are aggregated in specific environmental niches (river distance, paddy index), larval control could be considered as a supplemental measure to insecticide-treated nets or indoor residual spraying [54]. Further study assessing how few, fixed and findable larval sites are would help to determine the applicability of this approach.

Although we present results from mixed effects models with socioeconomic, environmental and climatic factors simultaneously, future research could take into account malaria-control interventions to model the transmission mechanism more accurately. For example, mathematical [55] and agent-based models [56] have been used to estimate the effects of malaria-control interventions. One benefit of these models is that they can model basic behavior of individual mosquitoes (including interactions within agents and to their environment [55, 56]), but dozens of simulation parameters must be available. A continuous surface of *An. sinensis* biting rates could be created by using Bayesian statistical framework in future studies, to provide a rational basis for control and spatial targeting [56, 58]. Maps of *An. sinensis* human biting rates may be very useful in vector management, but also could be used to generate a malaria risk map.

## Conclusion

Overall, our study found substantial spatial-temporal variation in mosquito human biting rates, which may help to explain the observed heterogeneity of malaria incidence in the surveillance regions. The temporal variation in *An. sinensis* human biting rate was mainly attributed to MT and EVI, while the most spatial variation in *An. sinensis* human biting rate resulted from river distance and paddy index. Continued entomologic monitoring to better understand the spatial-temporal variations of *An. sinensis* human biting rate will be vital to targeting vector control approaches to high risk areas and appropriate times of the year. More efficient targeting, supplemented with larval control activities where appropriate, may be a cost-effective approach for the ongoing malaria elimination program in China.

## Supporting Information

S1 Dataset Relevant data in Excel format. (XLSX)
